# Ethanol Upregulates NMDA Receptor Subunit Gene Expression in Human Embryonic Stem Cell-Derived Cortical Neurons

**DOI:** 10.1371/journal.pone.0134907

**Published:** 2015-08-12

**Authors:** Yangfei Xiang, Kun-Yong Kim, Joel Gelernter, In-Hyun Park, Huiping Zhang

**Affiliations:** 1 Department of Genetics, Yale University School of Medicine, New Haven, CT, United States of America; 2 Department of Psychiatry, Yale University School of Medicine, New Haven, CT, United States of America; 3 Department of Neurobiology, Yale University School of Medicine, New Haven, CT, United States of America; 4 VA Medical Center, VA Connecticut Healthcare System, West Haven, CT, United States of America; Vanderbilt University Medical Center, UNITED STATES

## Abstract

Chronic alcohol consumption may result in sustained gene expression alterations in the brain, leading to alcohol abuse or dependence. Because of ethical concerns of using live human brain cells in research, this hypothesis cannot be tested directly in live human brains. In the present study, we used human embryonic stem cell (hESC)-derived cortical neurons as *in vitro* cellular models to investigate alcohol-induced expression changes of genes involved in alcohol metabolism (*ALDH2*), anti-apoptosis (*BCL2* and *CCND2*), neurotransmission (NMDA receptor subunit genes: *GRIN1*, *GRIN2A*, *GRIN2B*, and *GRIN2D*), calcium channel activity (*ITPR2*), or transcriptional repression (*JARID2*). hESCs were differentiated into cortical neurons, which were characterized by immunostaining using antibodies against cortical neuron-specific biomarkers. Ethanol-induced gene expression changes were determined by reverse-transcription quantitative polymerase chain reaction (RT-qPCR). After a 7-day ethanol (50 mM) exposure followed by a 24-hour ethanol withdrawal treatment, five of the above nine genes (including all four NMDA receptor subunit genes) were highly upregulated (*GRIN1*: 1.93-fold, *P* = 0.003; *GRIN2A*: 1.40-fold, *P* = 0.003; *GRIN2B*: 1.75-fold, *P* = 0.002; *GRIN2D*: 1.86-fold, *P* = 0.048; *BCL2*: 1.34-fold, *P* = 0.031), and the results of *GRIN1*, *GRIN2A*, and *GRIN2B* survived multiple comparison correction. Our findings suggest that alcohol responsive genes, particularly NMDA receptor genes, play an important role in regulating neuronal function and mediating chronic alcohol consumption-induced neuroadaptations.

## Introduction

Alcohol use disorders (AUDs), including alcohol abuse and dependence, are common and complex genetic disorders, affecting about 8% of adult Americans each year [1] and causing substantial morbidity and mortality. Genetic association studies, including genome-wide association studies, have shown that many variants in numerous genes contribute to the risk of developing AUDs [2]. Moreover, chronic alcohol consumption could also lead to AUDs by altering expression of specific genes in reward-related brain regions [3]. Additionally, heavy and long-term use of alcohol can harm tissues, organs, or body systems because alcohol and its metabolite acetaldehyde are toxic [4]. Alcohol consumption is often related to or comorbid with a number of diseases such as neuropsychiatric disorders, liver cirrhosis, cancers, cardiovascular diseases, and infectious diseases [5].

To understand the mechanisms of AUDs and alcohol-related diseases, it is necessary to investigate alcohol-induced gene expression changes. It would be particularly informative to analyze gene expression changes in the brains of subjects affected with AUDs or alcohol-related diseases, but this is experimentally highly challenging. Due to ethical concerns of using live human brain tissues or neurons for research, most published studies have used cell lines, animal models, or postmortem human brain tissues to analyze alcohol exposure or consumption-induced expression changes of genes participating in alcohol metabolism, neurotransmission, neurotoxicity, intracellular calcium homeostasis, or transcriptional regulation.

Previous studies have revealed that alcohol consumption could alter the expression of alcohol-metabolizing genes, particularly the aldehyde dehydrogenase 2 (ALDH2) gene (*ALDH2*). Acetaldehyde, the major ethanol metabolite that is toxic and responsible for alcohol-induced tissue and cell injury, as well as flushing and other subjectively unpleasant sensations, is converted to acetic acid mainly by ALDH2. The expression and enzyme activity of ALDH2 can dramatically influence the vulnerability of individuals to AUDs [6,7]. Alcohol-induced upregulation of *ALDH2* expression has been observed in several studies. For example, preexposure of C57BL/6J mice to ethanol led to increased activity of ALDH2 [8]. Elevated expression levels of *ALDH2* mRNA were observed in human peripheral blood leukocytes after alcohol ingestion [9]. In our recent postmortem brain study, we reported that several alcohol-metabolizing genes including *ALDH2* were upregulated in the prefrontal cortex (PFC) of AUD subjects [10]. Additionally, Li et al. found that transgenic overexpression of *ALDH2* could effectively prevent acetaldehyde-induced cell injury [11]. These results indicate that *ALDH2* is a potential therapeutic target for the prevention and treatment of AUDs and alcohol-related disorders.

Long-term alcohol exposure alters the expression of genes involved in neurotransmission, leading to neuroadaptation to alcohol in the form of alcohol tolerance and dependence. N-methyl-D-aspartate (NMDA) receptors are a class of ionotropic glutamate receptors, and they play an essential role in synaptic transmission and plasticity as well as excitotoxicity [12,13]. They are the major targets of alcohol in the central nervous system and involved in ethanol-associated traits such as tolerance, dependence, withdrawal, craving, and relapse [14,15]. NMDA receptor channels are heterotetramers composed of two NR1 (or GluN1) and two NR2 (GluN2A-D) subunits [16] that surround a cation channel highly permeable to calcium ions [17]. Studies in animals have shown that both acute and chronic alcohol exposure affects the expression and activity of NMDA receptors. Acute alcohol exposure decreased NMDA excitatory postsynaptic potentials [18] and inhibited NMDA-dependent long-term potentiation [19–21]. However, chronic ethanol ingestion facilitated the expression of GluN1, GluN2A, and/or GluN2B subunits in rat cerebral cortex [22,23], amygdala [24], and hippocampus [23,25]. Therefore, expression alterations of NMDA receptor subunit genes due to chronic alcohol consumption may contribute to the development of AUDs.

Long-term exposure to alcohol can also lead to altered expression of genes involved in other biological pathways that are relevant to neurotoxicity, calcium ion transmembrane transport, or gene transcriptional regulation. The B-cell CLL/lymphoma 2 gene (*BCL2*) and the cyclin D2 gene (*CCND2*) are two anti-apoptotic genes. *BCL2* encodes an integral outer mitochondrial membrane protein that blocks the apoptotic death of cells and participates in the regulation of neural differentiation [26,27]. *CCND2* is a cell cycle regulatory gene that encodes cyclin D2 functioning in cell cycle G1/S transition and neurogenesis [28]. Yadav et al. reported that long-term ethanol exposure induced altered expression of *BCL2* and *CCND2* in human neuroblastoma cell line SH-SY5Y [29]. The Jumonji, AT rich interactive domain 2 gene (*JARID2*) encodes a DNA-binding protein that functions as a transcriptional repressor [30]. The inositol 1,4,5-trisphosphate receptor, type 2 gene (*ITPR2*) is involved in glutamate-mediated neurotransmission, intracellular calcium concentration regulation, and also plays an important role in apoptosis [31]. Recently, we found that the expression of both *JARID2* and *ITPR2* was upregulated in postmortem PFC of AUD subjects [10,32].

Although interesting findings have been generated from ethanol-exposed cells or animals or postmortem human brain tissues of AUD subjects, it is still unknown whether alcohol-induced gene expression changes reported in the above studies occur in live human brain neurons of AUD subjects. To model this living system, we used human embryonic stem cell (hESC)-derived cortical neurons *in vitro* to investigate chronic (defined, in this case, as one week) alcohol exposure-induced gene expression changes. We reported a significant upregulation of NMDA subunit gene expression in hESC-derived cortical neurons due to chronic ethanol exposure and ethanol withdrawal treatment.

## Materials and Methods

### Differentiation of human embryonic stem cells (hESCs) into cortical neurons

H1 hESCs (obtained from the WiCell Research Institute, Madison, USA) were differentiated into cortical neurons as previously described [33,34]. Briefly, H1 cells were dissociated with accutase and cultured on Matrigel coated plates with mTeSR1 medium (Stemcell technologies, Vancouver, Canada) for 4 days or until cells achieve over 95% confluence. Neural induction was initiated by culturing H1 cells in N3 medium containing TGF-β inhibitor (1 μM), retinoic acid (1 μM), and Noggin (500 ng/ml) for 8~11 days. Neuroepithelial cells were then isolated using dispase and replated into Ploy-L-laminin coated plates to induce the mature neuronal differentiation with N3 medium containing BDNF (10 ng/ml), GDNF (10 ng/ml), NT-3 (3 ng/ml), and CNTF (10 ng/ml). We also used Knockout Serum Replacer (KSR), N2 supplement, 100 nM LDN-193189, 10 μM SB-431542, and 2 μM XAV-939 to initiate differentiation for 10 days. N2 medium supplemented with B27 and containing 100 ng/mL SHH and 1 μM purmorphamine was then used for ventral patterning for eight days. After ventral patterning, medium was changed to neurobasal medium supplemented with B27 and containing 20 ng/mL BDNF, 200 μM ascorbic acid, and 200 μM cAMP for final differentiation and maturation. Neurons were differentiated for over two months before use.

### Immunostaining

H1 hESC-derived cortical neurons were characterized by immunostaining to confirm the expression of neuronal biomarkers as described previously [35]. Briefly, the differentiated neurons were fixed with 4% formaldehyde/PBS solution and then incubated with rabbit polyclonal antibody Tuj1 (Sigma-Aldrich, St. Louis, USA), which is specific for neuronal biomarker beta-III tubulin (a microtubule element of the tubulin family found almost exclusively in neurons). The expression of beta-III tubulin on the surface of hESC-differentiated neurons was visualized by incubation of the cells with Alexa Fluor 555-conjugated goat anti-rabbit secondary antibodies [36]. Similarly, the production of inhibitory neurotransmitter gamma-aminobutyric acid (GABA) by hESCs-derived neurons was detected by immunostaining using anti-GABA antibodies produced in rabbit (Sigma-Aldrich, St. Louis, USA).

### Chronic ethanol treatment and morphological examination

H1 ESC-derived cortical neurons were cultured in neuronal differentiation medium containing different concentrations of ethanol (0, 1, 5, 10, 50, and 100 mM) for seven days. The cultural medium was replaced every other day. Morphological changes of neurons due to ethanol exposure were investigated using fluorescence microscopy with the adoption of DAPI (4',6-diamidino-2-phenylindole) for chemical staining of DNA [37] and the Tuj1 antibody for immunostaining of neuronal biomarker beta-III tubulin [35].

### Determination of ethanol-induced gene expression changes using RT-qPCR

H1 hESC-derived neurons were used as *in vitro* cellular models for investigating ethanol-induced expression changes of nine candidate genes that participate in ethanol metabolism (*ALDH2*), glutamate neurotransmission (*GRIN1*, *GRIN2A*, *GRIN2B*, and *GRIN2D*), anti-apoptosis (*BCL2* and *CCND2*), calcium ion transmembrane transport activity (*ITPR2*), or gene transcriptional repression (*JARID2*). H1 hESC-derived neurons were cultured in differentiation medium containing 50 mM ethanol (equivalent to blood ethanol concentration in heavy drinkers [38,39]) for 7 days followed by a 24-hour withdrawal period. A sham treatment of H1 hESC-derived neurons with differentiation medium without ethanol was also carried out. Neuronal differentiation medium with or without 50 mM ethanol was replaced every other day. The experiments for both ethanol and sham treatments were performed in triplicate.

Total RNA was extracted from H1 hESC-derived cortical neurons (exposed or unexposed to 50 mM ethanol) using the RNeasy Mini Kit (QIAGEN, Valencia, USA). RNA samples were treated with RNase-Free DNase and purified with RNeasy mini columns (QIAGEN, Valencia, USA). cDNA was synthesized from 1 μg of RNA using the iScript Select cDNA Synthesis Kit (Bio-Rad, Hercules, USA). Expression levels of *ALDH2*, *GRIN1*, *GRIN2A*, *GRIN2B*, *GRIN2D*, *BCL2*, *CCND2*, *ITPR2*, and *JARID2* were analyzed using the SsoFast EvaGreen Supermix (Bio-Rad, Hercules, USA) in the CFX96 Real-Time PCR Detection System (Bio-Rad, Hercules, USA). The housekeeping gene *ACTIN* was used as the reference for estimating relative expression levels of target genes. PCR primers for the above 10 genes were designed using DNASTAR (SeqBuilder) software (http://www.dnastar.com), and they are listed in S1 **Table**. The cycling conditions were: 95°C for 15 min, followed by 40 two-step cycles at 94°C for 10 sec and 60°C for 45 sec. The threshold cycle (Ct) of the nine target genes and the reference gene (*ACTIN*) for each sample was analyzed using the software provided along with the CFX96 Real-Time PCR Detection System. The relative expression levels (ΔCt) of the above nine target genes were normalized to that of the reference gene *ACTIN* (ΔCt = Ct_Target gene_ – Ct_Actin_). A t-test was used to compare gene expression differences between neurons that were exposed and unexposed to ethanol. The relative quantity (RQ) of target gene expression in ethanol-exposed neurons in comparison to ethanol-unexposed neurons was calculated by formula: RQ = 2^-ΔΔCt^ [ΔΔCt = ΔCt_(ethanol-exposed)_ – ΔCt_(ethanol-unexposed)_].

## Results

**Fig 1:**
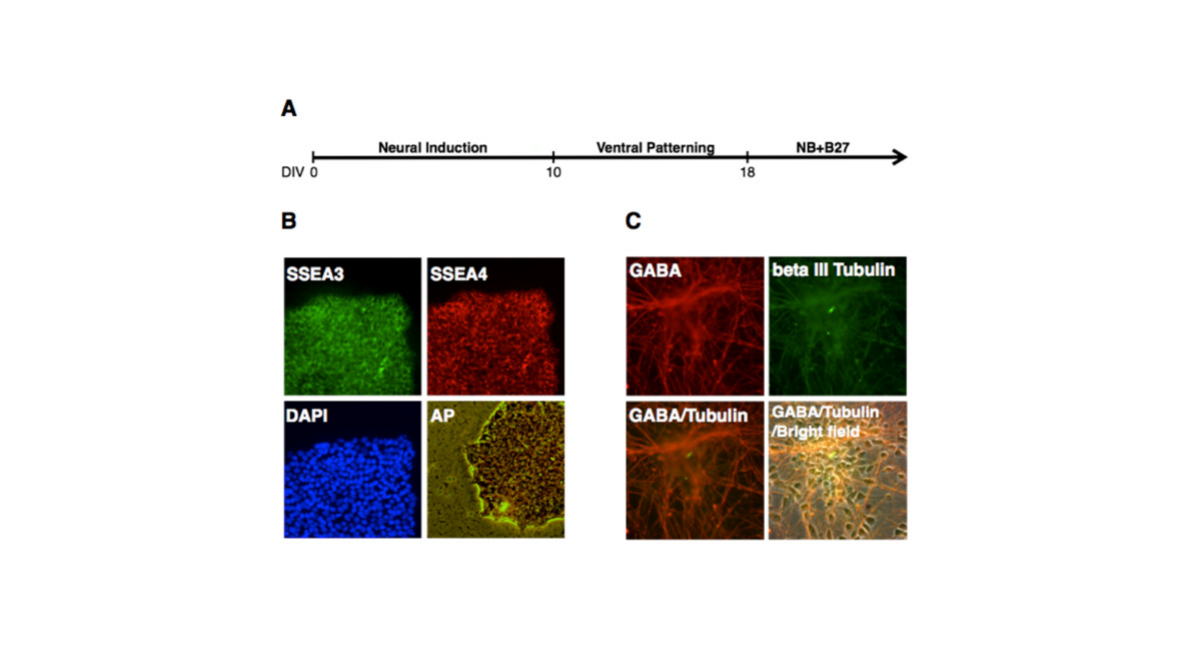
Neuronal differentiation of H1 hESCs. A. Schematic diagram for differentiating H1 hESCs into cortical neurons. B. Immunostaining [using antibodies reacting with stage-specific embryonic antigen-3 (SSEA3) and antigen-4 (SSEA-4)], nuclear staining [using 4',6-diamidino-2-phenylindole (DAPI) which is a fluorescent stain that binds strongly to A-T rich regions in DNA], and alkaline phosphatase (AP) staining (using a fluorescent substrate for AP for characterizing pluripotent stem cells) of H1 colonies before neuronal differentiation. C. Immunostaining of hESCs-derived neurons at days in dish (DIV) 32 using antibodies reacting with GABA and beta III tubulin, which are the biomarkers for GABA neurons.

hESC-derived neurons at days in dish (DIV) 32 were characterized by immunostaining of γ-aminobutyric acid (or GABA, i.e., the inhibitory neurotransmitter mainly produced by GABAergic neurons, which are cortical neurons in the mammalian central nervous system) and beta-III tubulin (a biomarker for mature neurons). As shown in **Fig 1**, hESC-derived neurons expressed both GABA and beta-III tubulin. Nevertheless, three stem cell-specific biomarkers [i.e., stage-specific embryonic antigen-3 (SSEA3) and antigen-4 (SSEA-4) as well as alkaline phosphatase (AP)] were not detectable in hESc-derived neurons. These results suggest that H1 ESCs were differentiated into cortical neurons.

**Fig 2:**
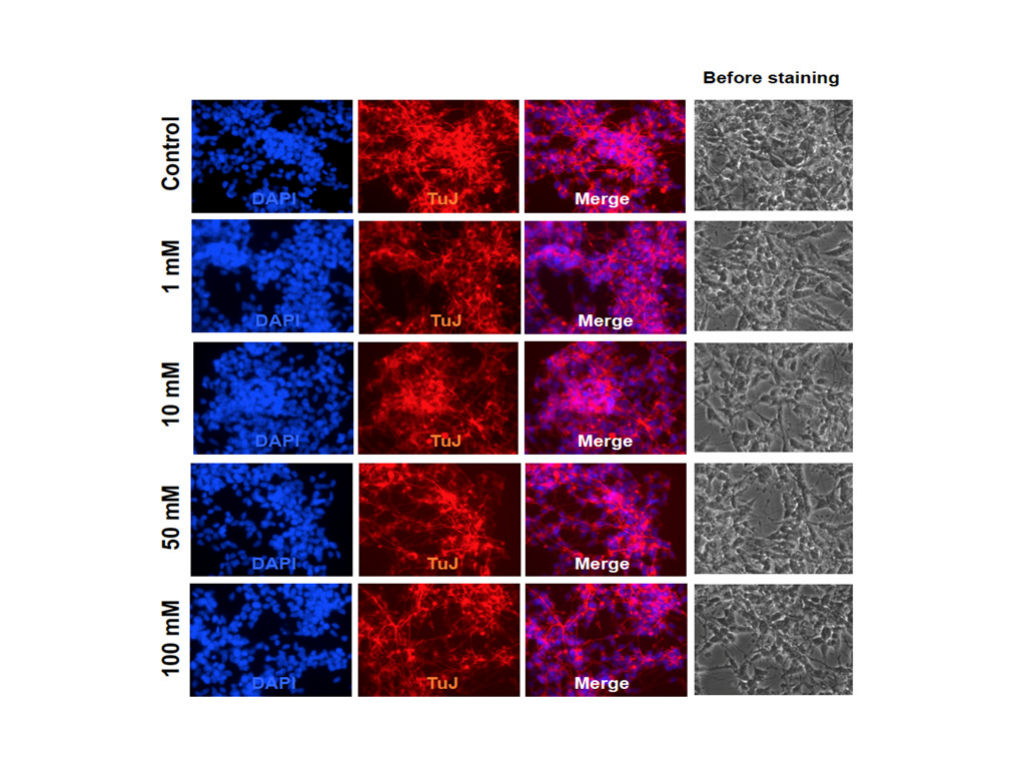
Morphological changes of H1 hESC-derived cortical neurons exposed to ethanol. Morphological changes of H1 hESC-derived cortical neurons exposed to different concentrations of ethanol (0, 1, 10, 50, and 100 mM) were examined by DAPI DNA staining and Tuj1 (antibody specific for neuronal marker beta-III tubulin) immunostaining.

**Fig 3:**
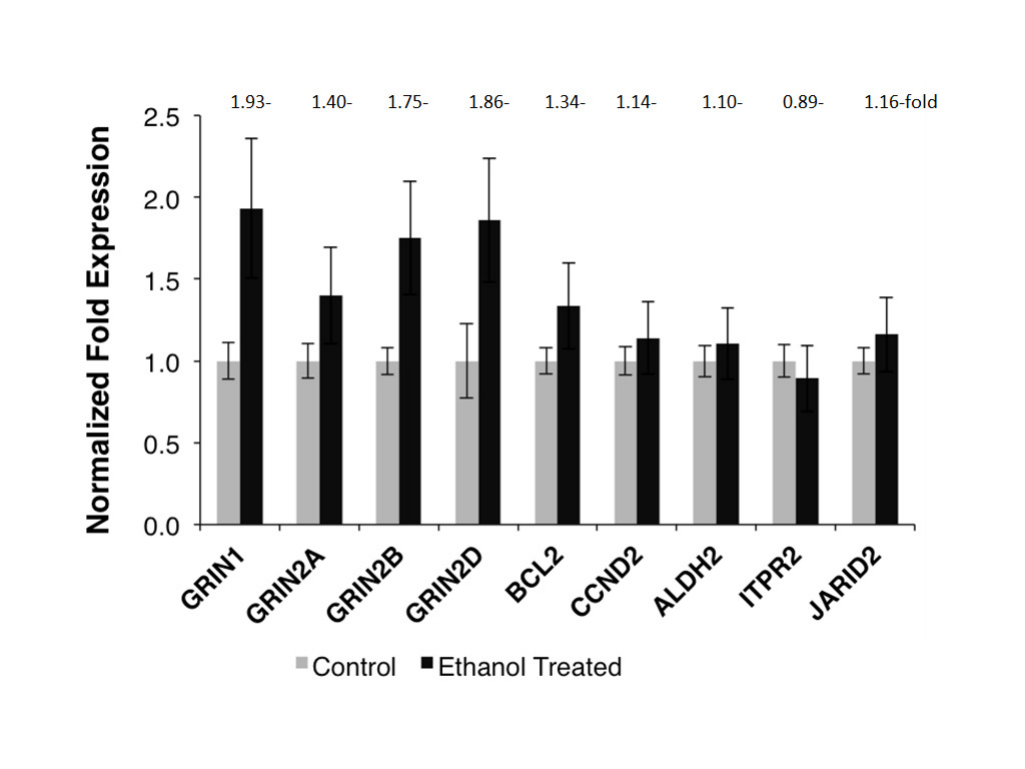
Effects of ethanol on gene expression in cortical neurons differentiated from H1 hESCs. Expression changes of nine genes in H1 hESC differentiated cortical neurons exposed to 50 mM ethanol for 7 days plus 24-hr ethanol withdrawal treatment were determined by RT-qPCR. Left column for each gene: without ethanol exposure; right column for each gene: with ethanol exposure. The expression level of each gene in ethanol-unexposed neurons was normalized to “1”. *GRIN1*, *2A*, *2B*, and *2D*: the NMDA receptor subunit genes; *BCL2*: the B-cell CLL/lymphoma 2 gene; *CCND2*: the cyclin D2 gene; *ALDH2*: the aldehyde dehydrogenase 2 gene; *ITPR2*: the inositol 1,4,5-trisphosphate receptor (type 2) gene; and *JARID2*: the jumonji- and AT-rich interactive domain 2 gene. The results were representative of three separate experiments. Each value represented the mean ± SD of triplicate wells.

Exposure of hESC-derived neurons to ethanol at concentrations of up to 100 mM for seven days did not cause apparent morphological changes or cell death (**Fig 2**). We further examined ethanol-induced gene expression changes in hESC-derived neurons that were exposed to 50 mM ethanol for seven days and followed by a 24-hour ethanol withdrawal treatment. As shown in **Fig 3,** ethanol exposure and withdrawal resulted in upregulation of all four NMDA receptor subunit genes involved in excitatory glutamatergic neurotransmission (*GRIN1*: 1.93-fold, *P* = 0.003; *GRIN2A*: 1.40-fold, *P* = 0.003; *GRIN2B*: 1.75-fold, *P* = 0.002; *GRIN2D*: 1.86-fold, *P* = 0.048). Additionally, the expression level of the anti-apoptotic gene *BCL2* was also significantly increased in hESC-derived neurons receiving ethanol exposure and withdrawal treatment (1.34-fold, *P* = 0.031). Nevertheless, four other genes did not show significant expression changes in hESC-derived neurons after ethanol exposure and withdrawal treatment (*ALDH2*: 1.10-fold, *P* = 0.100; *CCND2*: 1.14-fold, *P* = 0.057; *JARID2*: 1.16-fold, *P* = 0.079; *ITPR2*: 0.89-fold, *P* = 0.250). After the Bonferroni adjustment (α = 0.05/9 = 0.006), the results of three NMDA receptor subunit genes (*GRIN1*, *GRIN2A*, and *GRIN2B*) remained significant.

## Discussion

This pilot study explored the possibility of using hESC-derived neurons as *in vitro* cellular models to examine ethanol-induced alterations in the morphology of neurons and the expression of genes participating in ethanol metabolism, neurotransmission, neuronal apoptosis, calcium ion transmembrane transport, and gene transcription regulation. Although exposure of hESC-derived neurons to ethanol at concentrations of up to 100 mM did not cause cell death or apparent morphological changes (similar results were observed in ethanol-exposed human epithelial cells [40] and zebrafish brain neurons [41]), chronic ethanol exposure in combination with ethanol withdrawal did alter expression of specific genes involved in pathways for developing AUDs.

Our study demonstrated that the expression levels of NMDA receptor subunit genes (*GRIN1*, *GRIN2A*, *GRIN2B*, and *GRIN2D*) were significantly upregulated in hESC-derived neurons that experienced chronic ethanol exposure and ethanol withdrawal treatment. Our findings are consistent with those from studies in animal models [22–25]. Our study also confirmed the research results reported by Lieberman et al. [42], who analyzed the effects of ethanol exposure and withdrawal on NMDA receptor subunit gene expression in human induced pluripotent stem cell (iPSC)-derived neurons. Contrary to the findings from the present study, a postmortem study by Ridge et al. [43] showed that the expression levels of *GRIN1*, *GRIN2A*, and *GRIN2B* mRNAs were lowered in both superior frontal and primary motor cortex tissues of alcoholic subjects with liver cirrhosis in comparison to nonalcoholic control subjects or alcoholic subjects without comorbid disorders. Moreover, in our recent postmortem brain study using PFC tissues of alcoholic patients and matched control subjects, we did not observe AUD-associated expression changes of *GRIN1*, *GRIN2A*, *GRIN2B*, and *GRIN2D* mRNAs [10]. Although studies using postmortem human brain tissues can circumvent the concerns regarding the dissimilarity of brain gene expression patterns between humans and animals, some confounding factors, such as sex, age, amount of alcohol consumption, postmortem interval, comorbid drug addiction or diseases, and/or medications, may bias the findings. In the present study, we employed hESC-derived neurons to address this issue, and we assume that ethanol-induced gene expression changes in hESC-derived neurons is more likely to reflect those changes appeared in brain neurons of human alcoholic subjects. Certainly, the regulation of NMDA receptor subunit gene expression in the brain is highly complex; their transcription is under the control of both genetic and non-genetic factors. Several studies have provided evidence that acute and chronic ethanol exposure exerts opposite effects on NMDA receptor subunit gene expression as well as NMDA receptor-dependent glutamatergic transmission and long-term potentiation. Acute ethanol exposure inhibits the expression and function of NMDA receptors [18,19], whereas prolonged ethanol exposure leads to a compensatory “upregulation” of NMDA receptor-mediated functions even after removal of ethanol [44]. The enhanced expression and function of NMDA receptors may contribute to chronic ethanol consumption-induced neuroadaptations.

The present study also showed that ethanol exposure and withdrawal might result in altered expression of several other genes participating in cellular functions such as ethanol metabolism, anti-apoptosis, calcium ion channel activity, or gene transcription; however, the extent of expression changes of these genes was not as great as what was observed in NMDA receptor subunit genes. Since *ALDH2* is the major gene for converting toxic acetaldehyde to non-toxic acetate, and two anti-apoptotic genes (*BCL2* and *CCND2*) are potentially involved in protecting cells from acetaldehyde-caused neurotoxicity, the slightly elevated expression levels of these three genes (*ALDH2*: 1.10-fold; *BCL2*: 1.34-fold; *CCND2*: 1.14-fold) after ethanol exposure and withdrawal (**Fig 3**) might be beneficial for the function of neurons. We also observed a slightly increased expression of *JARID2* (1.16-fold) in hESC-derived neurons that were treated with ethanol exposure and withdrawal, but it is unclear how expression changes of this transcriptional repressor gene influence the activity of neurons or ethanol-induced neuroadaptations. Ethanol can regulate the function of voltage-dependent calcium ion channels [45], but the mechanism of this regulation is not well studied. Possibly, ethanol-induced expression changes of genes such as *ITPR2* (involved in calcium ion transmembrane transport) could moderate the activity of calcium ion channels. In our recent postmortem brain study, mRNA expression levels of *JARID2* and *ITPR2* were both upregulated in postmortem PFC of AUD subjects [10,32]. Hence, we expected that ethanol could induce upregulation of *ITPR2* in hESC-derived neurons. Beyond of our expectation, a slightly decreased expression of *ITPR2* mRNA (0.89-fold) was observed in neurons with ethanol exposure and withdrawal treatment. Previous studies showed that acute exposure of neurons to ethanol produced a concentration-dependent decrease in depolarization-evoked calcium uptake, while prolonged exposure led to an increase in calcium uptake, and calcium uptake was restored to control levels following withdrawal of ethanol from culture medium [46]. Therefore, the slight downregulation of *ITPR2* observed in our study was likely due to withdrawal of ethanol for 24 hours following a 7-day ethanol exposure. Taken together, the present study using neuronal models provided additional information for understanding the molecular mechanisms of AUDs.

Although the application of hESC-derived neurons in the study of ethanol-induced gene expression alterations is a novel approach for understanding neuroadaptations resulting from chronic alcohol consumption, several issues still remain to be addressed in future studies. First, we examined alcohol-induced expression changes of only several candidate genes involved in a limited number of biological pathways relevant to AUDs or neuronal functions. Future studies should use expression microarray or RNA sequencing approach to analyze genome-wide gene expression changes caused by chronic ethanol exposure and withdrawal. Second, the mechanisms by which ethanol induces gene expression changes are unknown. More evidence suggests that ethanol exposure may result in chromatin remodeling *via* covalent histone modifications and DNA methylation, leading to gene expression changes [47,48]. Recently, we examined DNA methylation changes in the promoter region of the serotonin receptor 3a gene (*Htr3a*) using the drinking-in-the-dark CD-1 mouse model and found that ethanol could induce DNA methylation and correlated gene expression changes in specific brain regions [49,50]. In our postmortem brain study, we identified a differentially expressed microRNA miR-130a in the PFC of AUD subjects, and expression levels of miR-130a and several predicted target genes (including *JARID2* and *ITPR2*) were negatively correlated [32], indicating ethanol-induced gene expression changes may also be mediated by microRNA regulatory pathways. Therefore, in our follow-up studies, ethanol exposure and withdrawal-induced epigenetic changes that may lead to altered gene expression should be analyzed. Third, we only reported ethanol-induced molecular changes at the mRNA level; further studies are needed to investigate whether ethanol exposure and withdrawal cause cellular activity changes (e.g., using patch-clamp or calcium imaging to determine ethanol-induced calcium ion channel activity changes). Finally, the definition of one week of exposure to 50 mM ethanol as “chronic” is arbitrary. It is necessary to investigate gene expression changes at different time points during ethanol exposure and withdrawal periods.

In summary, the findings from the present study suggest that hESC-derived neurons are useful cellular models for examining the molecular mechanisms underlying alcohol-induced neuroadaptations. hESC-derived neurons could also be used to study the interactive effects of genetic variation and ethanol exposure/withdrawal on molecular and cellular functions of neuronal cells. Furthermore, it is our expectation that hESC-derived neurons are useful tools for screening novel and less-toxic medications for treatment of AUDs and related diseases.

## Supporting Information

S1 Table Primers for measuring gene expression by real-time PCR and PCR product sizes. (DOCX)

## References

[1] Grant BF, Dawson DA, Stinson FS, Chou SP, Dufour MC, Pickering RP (2004) The 12-month prevalence and trends in DSM-IV alcohol abuse and dependence: United States, 1991–1992 and 2001–2002. Drug Alcohol Depend 74: 223–234. 15194200 10.1016/j.drugalcdep.2004.02.004

[2] Edenberg HJ, Foroud T (2013) Genetics and alcoholism. Nat Rev Gastroenterol Hepatol 10: 487–494. 10.1038/nrgastro.2013.86 23712313 PMC4056340

[3] Hansson AC, Rimondini R, Neznanova O, Sommer WH, Heilig M (2008) Neuroplasticity in brain reward circuitry following a history of ethanol dependence. Eur J Neurosci 27: 1912–1922. 10.1111/j.1460-9568.2008.06159.x 18412612 PMC2486413

[4] Melgaard B (1983) The neurotoxicity of ethanol. Acta Neurol Scand 67: 131–142. 6135290 10.1111/j.1600-0404.1983.tb04556.x

[5] Corrao G, Bagnardi V, Zambon A, La Vecchia C (2004) A meta-analysis of alcohol consumption and the risk of 15 diseases. Prev Med 38: 613–619. 15066364 10.1016/j.ypmed.2003.11.027

[6] Li D, Zhao H, Gelernter J (2012) Strong protective effect of the aldehyde dehydrogenase gene (ALDH2) 504lys (*2) allele against alcoholism and alcohol-induced medical diseases in Asians. Hum Genet 131: 725–737. 10.1007/s00439-011-1116-4 22102315 PMC3548401

[7] Quillen EE, Chen XD, Almasy L, Yang F, He H, Li X, et al (2014) ALDH2 is associated to alcohol dependence and is the major genetic determinant of "daily maximum drinks" in a GWAS study of an isolated rural chinese sample. Am J Med Genet B Neuropsychiatr Genet 165B: 103–110. 10.1002/ajmg.b.32213 24277619 PMC4149216

[8] Ding X, Beier JI, Baldauf KJ, Jokinen JD, Zhong H, Arteel GE (2014) Acute ethanol preexposure promotes liver regeneration after partial hepatectomy in mice by activating ALDH2. Am J Physiol Gastrointest Liver Physiol 306: G37–47. 10.1152/ajpgi.00085.2013 24177029 PMC3920082

[9] Kimura Y, Nishimura FT, Abe S, Fukunaga T, Tanii H, Saijoh K (2009) A promoter polymorphism in the ALDH2 gene affects its basal and acetaldehyde/ethanol-induced gene expression in human peripheral blood leukocytes and HepG2 cells. Alcohol Alcohol 44: 261–266. 10.1093/alcalc/agn123 19144977

[10] Zhang H, Wang F, Xu H, Liu Y, Liu J, Zhao H, et al (2014) Differentially co-expressed genes in postmortem prefrontal cortex of individuals with alcohol use disorders: influence on alcohol metabolism-related pathways. Hum Genet 133: 1383–1394. 10.1007/s00439-014-1473-x 25073604 PMC4185230

[11] Li SY, Gomelsky M, Duan J, Zhang Z, Gomelsky L, Zhang X, et al (2004) Overexpression of aldehyde dehydrogenase-2 (ALDH2) transgene prevents acetaldehyde-induced cell injury in human umbilical vein endothelial cells: role of ERK and p38 mitogen-activated protein kinase. J Biol Chem 279: 11244–11252. 14722101 10.1074/jbc.M308011200

[12] Bliss TV, Collingridge GL (1993) A synaptic model of memory: long-term potentiation in the hippocampus. Nature 361: 31–39. 8421494 10.1038/361031a0

[13] Chandler LJ, Newsom H, Sumners C, Crews F (1993) Chronic ethanol exposure potentiates NMDA excitotoxicity in cerebral cortical neurons. J Neurochem 60: 1578–1581. 8455043 10.1111/j.1471-4159.1993.tb03326.x

[14] Trujillo KA, Akil H (1995) Excitatory amino acids and drugs of abuse: a role for N-methyl-D-aspartate receptors in drug tolerance, sensitization and physical dependence. Drug Alcohol Depend 38: 139–154. 7671766 10.1016/0376-8716(95)01119-j

[15] Krystal JH, Petrakis IL, Mason G, Trevisan L, D'Souza DC (2003) N-methyl-D-aspartate glutamate receptors and alcoholism: reward, dependence, treatment, and vulnerability. Pharmacol Ther 99: 79–94. 12804700 10.1016/s0163-7258(03)00054-8

[16] Bigge CF (1999) Ionotropic glutamate receptors. Curr Opin Chem Biol 3: 441–447. 10419857 10.1016/S1367-5931(99)80065-9

[17] Wu HY, Yuen EY, Lu YF, Matsushita M, Matsui H, Yan Z, et al (2005) Regulation of N-methyl-D-aspartate receptors by calpain in cortical neurons. J Biol Chem 280: 21588–21593. 15790561 10.1074/jbc.M501603200

[18] Nie Z, Madamba SG, Siggins GR (1994) Ethanol inhibits glutamatergic neurotransmission in nucleus accumbens neurons by multiple mechanisms. J Pharmacol Exp Ther 271: 1566–1573. 7527857

[19] Puglia MP, Valenzuela CF (2010) Ethanol acutely inhibits ionotropic glutamate receptor-mediated responses and long-term potentiation in the developing CA1 hippocampus. Alcohol Clin Exp Res 34: 594–606. 10.1111/j.1530-0277.2009.01128.x 20102565 PMC3050571

[20] Hicklin TR, Wu PH, Radcliffe RA, Freund RK, Goebel-Goody SM, Correa PR, et al (2011) Alcohol inhibition of the NMDA receptor function, long-term potentiation, and fear learning requires striatal-enriched protein tyrosine phosphatase. Proc Natl Acad Sci U S A 108: 6650–6655. 10.1073/pnas.1017856108 21464302 PMC3081035

[21] Mishra D, Zhang X, Chergui K (2012) Ethanol disrupts the mechanisms of induction of long-term potentiation in the mouse nucleus accumbens. Alcohol Clin Exp Res 36: 2117–2125. 10.1111/j.1530-0277.2012.01824.x 22551245

[22] Chandler LJ, Norwood D, Sutton G (1999) Chronic ethanol upregulates NMDA and AMPA, but not kainate receptor subunit proteins in rat primary cortical cultures. Alcohol Clin Exp Res 23: 363–370. 10069569

[23] Follesa P, Ticku MK (1995) Chronic ethanol treatment differentially regulates NMDA receptor subunit mRNA expression in rat brain. Brain Res Mol Brain Res 29: 99–106. 7770006 10.1016/0169-328x(94)00235-7

[24] Floyd DW, Jung KY, McCool BA (2003) Chronic ethanol ingestion facilitates N-methyl-D-aspartate receptor function and expression in rat lateral/basolateral amygdala neurons. J Pharmacol Exp Ther 307: 1020–1029. 14534353 10.1124/jpet.103.057505

[25] Maler JM, Esselmann H, Wiltfang J, Kunz N, Lewczuk P, Reulbach U, et al (2005) Memantine inhibits ethanol-induced NMDA receptor up-regulation in rat hippocampal neurons. Brain Res 1052: 156–162. 16009352 10.1016/j.brainres.2005.06.017

[26] Zhang KZ, Westberg JA, Holtta E, Andersson LC (1996) BCL2 regulates neural differentiation. Proc Natl Acad Sci U S A 93: 4504–4508. 8633098 10.1073/pnas.93.9.4504 PMC39568

[27] Hui K, Kucera J, Henderson JT (2008) Differential sensitivity of skeletal and fusimotor neurons to Bcl-2-mediated apoptosis during neuromuscular development. Cell Death Differ 15: 691–699. 18097449 10.1038/sj.cdd.4402294

[28] Kowalczyk A, Filipkowski RK, Rylski M, Wilczynski GM, Konopacki FA, Jaworski J, et al (2004) The critical role of cyclin D2 in adult neurogenesis. J Cell Biol 167: 209–213. 15504908 10.1083/jcb.200404181 PMC2172537

[29] Yadav S, Pandey A, Shukla A, Talwelkar SS, Kumar A, et al (2011) miR-497 and miR-302b regulate ethanol-induced neuronal cell death through BCL2 protein and cyclin D2. J Biol Chem 286: 37347–37357. 10.1074/jbc.M111.235531 21878650 PMC3199482

[30] Escobar TM, Kanellopoulou C, Kugler DG, Kilaru G, Nguyen CK, Nagarajan V, et al (2014) miR-155 activates cytokine gene expression in Th17 cells by regulating the DNA-binding protein Jarid2 to relieve polycomb-mediated repression. Immunity 40: 865–879. 10.1016/j.immuni.2014.03.014 24856900 PMC4092165

[31] van Es MA, Van Vught PW, Blauw HM, Franke L, Saris CG, Andersen PM, et al (2007) ITPR2 as a susceptibility gene in sporadic amyotrophic lateral sclerosis: a genome-wide association study. Lancet Neurol 6: 869–877. 17827064 10.1016/S1474-4422(07)70222-3

[32] Wang F, Gelernter J, Zhang H (2013) Differential Expression of miR-130a in Postmortem Prefrontal Cortex of Subjects with Alcohol Use Disorders. J Addict Res Ther 4. 10.4172/2155-6105.1000155 PMC4221234 25383235

[33] Maroof AM, Keros S, Tyson JA, Ying SW, Ganat YM, Merkle FT, et al (2013) Directed differentiation and functional maturation of cortical interneurons from human embryonic stem cells. Cell Stem Cell 12: 559–572. 10.1016/j.stem.2013.04.008 23642365 PMC3681523

[34] Shi Y, Kirwan P, Smith J, Robinson HP, Livesey FJ (2012) Human cerebral cortex development from pluripotent stem cells to functional excitatory synapses. Nat Neurosci 15: 477–486. 10.1038/nn.3041 22306606 PMC3882590

[35] Kim KY, Hysolli E, Park IH (2011) Neuronal maturation defect in induced pluripotent stem cells from patients with Rett syndrome. Proc Natl Acad Sci U S A 108: 14169–14174. 10.1073/pnas.1018979108 21807996 PMC3161557

[36] Ma L, Hu B, Liu Y, Vermilyea SC, Liu H, Gao L, et al (2012) Human embryonic stem cell-derived GABA neurons correct locomotion deficits in quinolinic acid-lesioned mice. Cell Stem Cell 10: 455–464. 10.1016/j.stem.2012.01.021 22424902 PMC3322292

[37] Kapuscinski J (1995) DAPI: a DNA-specific fluorescent probe. Biotech Histochem 70: 220–233. 8580206 10.3109/10520299509108199

[38] Hamlyn AN, Brown AJ, Sherlock S, Baron DN (1975) Casual blood-ethanol estimations in patients with chronic liver disease. Lancet 2: 345–347. 51146 10.1016/s0140-6736(75)92781-6

[39] Jones AW, Lowinger H (1988) Relationship between the concentration of ethanol and methanol in blood samples from Swedish drinking drivers. Forensic Sci Int 37: 277–285. 3410397 10.1016/0379-0738(88)90236-8

[40] Guo W, Baluda MA, Park NH (1997) Ethanol upregulates the expression of p21 WAF1/CIP1 and prolongs G1 transition via a p53-independent pathway in human epithelial cells. Oncogene 15: 1143–1149. 9294607 10.1038/sj.onc.1201287

[41] Puttonen HA, Sundvik M, Rozov S, Chen YC, Panula P (2013) Acute ethanol treatment upregulates Th1, Th2, and Hdc in larval zebrafish in stable networks. Front Neural Circuits 7: 102 10.3389/fncir.2013.00102 23754986 PMC3668275

[42] Lieberman R, Levine ES, Kranzler HR, Abreu C, Covault J (2012) Pilot study of iPS-derived neural cells to examine biologic effects of alcohol on human neurons in vitro. Alcohol Clin Exp Res 36: 1678–1687. 10.1111/j.1530-0277.2012.01792.x 22486492 PMC3424319

[43] Ridge JP, Ho AM, Innes DJ, Dodd PR (2008) The expression of NMDA receptor subunit mRNA in human chronic alcoholics. Ann N Y Acad Sci 1139: 10–19. 10.1196/annals.1432.053 18991843 PMC3821866

[44] Nagy J (2008) Alcohol related changes in regulation of NMDA receptor functions. Curr Neuropharmacol 6: 39–54. 10.2174/157015908783769662 19305787 PMC2645546

[45] Lancaster FE (1992) Alcohol, nitric oxide, and neurotoxicity: is there a connection?—a review. Alcohol Clin Exp Res 16: 539–541. 1320808 10.1111/j.1530-0277.1992.tb01413.x

[46] Messing RO, Carpenter CL, Diamond I, Greenberg DA (1986) Ethanol regulates calcium channels in clonal neural cells. Proc Natl Acad Sci U S A 83: 6213–6215. 2426713 10.1073/pnas.83.16.6213 PMC386470

[47] Starkman BG, Sakharkar AJ, Pandey SC (2012) Epigenetics-beyond the genome in alcoholism. Alcohol Res 34: 293–305. 23134045 10.35946/arcr.v34.3.04 PMC3860414

[48] Harlaar N, Hutchison KE (2013) Alcohol and the methylome: design and analysis considerations for research using human samples. Drug Alcohol Depend 133: 305–316. 10.1016/j.drugalcdep.2013.07.026 23968814

[49] Barker JM, Zhang Y, Wang F, Taylor JR, Zhang H (2013) Ethanol-induced Htr3a promoter methylation changes in mouse blood and brain. Alcohol Clin Exp Res 37 Suppl 1: E101–107. 10.1111/j.1530-0277.2012.01906.x 22834954 PMC3511914

[50] Barker JM, Zhang H, Villafane JJ, Wang TL, Torregrossa MM, Taylor JR (2014) Epigenetic and pharmacological regulation of 5HT3 receptors controls compulsive ethanol seeking in mice. Eur J Neurosci 39: 999–1008. 24772465 10.1111/ejn.12477 PMC4004969

