# Supplementary material for: Ethanol Upregulates NMDA Receptor Subunit Gene Expression in Human Embryonic Stem Cell-Derived Cortical Neurons
Source: PLoS One. 2015 Aug 12;10(8):e0134907. doi: 10.1371/journal.pone.0134907 (PMC4534442; doi:10.1371/journal.pone.0134907)
Supplement: S1 Table — (DOCX) [file pone.0134907.s001.docx]

**SUPPORTING INFORMATION**

**Table S1.** Primers for measuring gene expression by real-time PCR and PCR product sizes

Genes Primer sequences (5’→3’) PCR product sizes (bp)

*ACTIN* (Forward) GACAGGATGCAGAAGGAGA 138

*ACTIN* (Reverse) CCACATCTGCTGGAAGGTGG

*ALDH2* (Forward) ATCCCCATTGACGGAGACTTC 141

*ALDH2* (Reverse) CACAACCACGTTTCCAGTTGC

*GRIN1* (Forward) CTACCGCATACCCGTGCTG 128

*GRIN1* (Reverse) GCATCATCTCAAACCACACGC

*GRIN2A* (Forward) TGGCCTCACCGGGTATGATT 152

*GRIN2A* (Reverse) CAATGCCGTCCCTCACTCTC

*GRIN2B* (Forward) GTCCCTGGACGATGGAGATTC 131

*GRIN2B* (Reverse) CAGTCAGCCCTACTGAGTTGG

*GRIN2D* (Forward) GAGGAAAGGCCGTTTGTCATC 100

*GRIN2D* (Reverse) TGTGGGTTCGGTTGAGCTG

*BCL2* (Forward) GGTGGGGTCATGTGTGTGG 89

*BCL2* (Reverse) CGGTTCAGGTACTCAGTCATCC

*CCND2* (Forward) TTTGCCATGTACCCACCGTC 104

*CCND2* (Reverse) AGGGCATCACAAGTGAGCG

*JARID2* (Forward) AGCAACGTCATGATCTCCCC 191

*JARID2* (Reverse) GCGGTCTCAAAGCCCATACT

*ITPR2* (Forward) TCTTCAGCAACATCCAAAGCA 208

*ITPR2* (Reverse) AGAAGTTCTGCAATTCCCGGTT
